# Recent Advances in Single-Cell Profiling and Multispecific Therapeutics: Paving the Way for a New Era of Precision Medicine Targeting Cardiac Fibroblasts

**DOI:** 10.1007/s11886-021-01517-z

**Published:** 2021-06-03

**Authors:** Sally Yu Shi, Xin Luo, Tracy M. Yamawaki, Chi-Ming Li, Brandon Ason, Milena B. Furtado

**Affiliations:** 1 Department of Cardiometabolic Disorders, Amgen Discovery Research, Amgen Inc., 1120 Veterans Blvd, South San Francisco, CA 94080 USA; 2 Genome Analysis Unit, Amgen Discovery Research, Amgen Inc., 1120 Veterans Blvd, South San Francisco, CA 94080 USA

**Keywords:** Cardiac fibroblasts, Cardiac fibrosis, Heart failure, Single-cell RNA-sequencing, Multispecific therapeutics, Precision medicine

## Abstract

**Purpose of Review:**

Cardiac fibroblast activation contributes to fibrosis, maladaptive remodeling and heart failure progression. This review summarizes the latest findings on cardiac fibroblast activation dynamics derived from single-cell transcriptomic analyses and discusses how this information may aid the development of new multispecific medicines.

**Recent Findings:**

Advances in single-cell gene expression technologies have led to the discovery of distinct fibroblast subsets, some of which are more prevalent in diseased tissue and exhibit temporal changes in response to injury. In parallel to the rapid development of single-cell platforms, the advent of multispecific therapeutics is beginning to transform the biopharmaceutical landscape, paving the way for the selective targeting of diseased fibroblast subpopulations.

**Summary:**

Insights gained from single-cell technologies reveal critical cardiac fibroblast subsets that play a pathogenic role in the progression of heart failure. Combined with the development of multispecific therapeutic agents that have enabled access to previously “undruggable” targets, we are entering a new era of precision medicine.

## Introduction

Heart failure is the leading cause of morbidity and mortality around the world [1]. During development of chronic heart failure, excessive collagen deposition accumulates throughout the heart, resulting in interstitial fibrosis that contributes to stiffening of the heart, contractile dysfunction, and arrhythmias [2–4]. Interstitial fibrosis is primarily mediated by the activation of resident cardiac fibroblasts, which undergo phenotypic changes to adopt a secretory and pro-inflammatory cell state that communicates with and responds to other interstitial cells of the heart, in particular immune cells [2–4]. The classical activated state of a fibroblast, often referred to as a myofibroblast, represents a highly secretory cell type that produces fibrillar collagen and other extracellular matrix (ECM) proteins. Recent progress in the field has revealed that aberrant and sustained activation of fibroblasts fuels heart failure progression [4]. Consequently, targeting cardiac fibroblast activation represents a novel approach to reduce interstitial fibrosis and ameliorate heart failure.

Despite increased recognition of cardiac fibroblasts as a key driver in heart failure progression, current treatments for chronic heart failure do not specifically target fibroblasts. In this review, we will summarize recent research that highlights a pathogenic role of cardiac fibroblasts in heart failure and describe insights gained from single-cell RNA-sequencing (scRNA-seq) studies, focusing on those that have deepened our understanding of cardiac fibroblast phenotypic heterogeneity and dynamic cellular transitions in response to injury. In addition, we will discuss how these data can be leveraged for the selective targeting of disease-associated fibroblast subsets.

## Cardiac Fibroblast as a Driver in Heart Failure Progression

Historically, fibroblasts have been difficult to study due to limitations in the ability to unambiguously identify these cells and manipulate them in vivo. Advances in molecular genetic tools coupled with lineage tracing have allowed for a more precise evaluation of the embryonic origin and molecular identity of resident cardiac fibroblasts (reviewed in [5–8]). In parallel, recent work has led to a greater appreciation of their causal role in heart failure progression [9, 10]. For example, in a murine model of pressure overload–induced heart failure by transverse aortic constriction (TAC), myofibroblast-specific inhibition of transforming growth factor β (TGFβ) signaling attenuated cardiac fibrosis, providing evidence that activation of tissue-resident fibroblasts by TGFβ is responsible for the fibrotic response in the heart [11•]. In addition to signaling mediated by biochemical agents such as TGFβ, maintenance of a chronic fibrotic response depends on the biophysical microenvironment and mechanical properties of the cardiac ECM, whereby matrix stiffness promotes mechano-activation of fibroblasts and perpetuates a profibrotic milieu [12–14].

TGFβ is considered a master regulator of myofibroblast activation [15, 16]. In addition to canonical signaling through SMAD transcription factors [11•, 17], TGFβ signals through non-canonical pathways such as p38 MAPK [18, 19]. This redistributes the transcriptional coactivator BRD4, activating profibrotic gene expression [20]. In models of genetic cardiomyopathy driven by expression of a 40-kDa fragment of cardiac myosin binding protein C (cMyBP-C) [21•] or a mutant alphaB-crystallin (CryAB R120G) [22•], conditional deletion of TGFβ signaling in myofibroblasts reduced fibrosis, improved cardiac function, and increased the probability of survival. Notably, in both of these models, the primary defect was cardiomyocyte intrinsic, and activation of fibroblasts was a secondary response to myocyte stress [21•, 22•]. The fact that inhibiting secondary fibroblast activation conferred hemodynamic benefit and arrested adverse remodeling further corroborates the notion that myofibroblasts are a major disease driver in heart failure progression.

Nevertheless, despite the critical involvement of TGFβ signaling [10, 23] and other profibrotic pathways such as angiotensin II (Ang II) and endothelin 1 [24] in the development of cardiac fibrosis, therapeutic targeting of these pathways has had limited success in fibrosis amelioration. Treatment with the renin-angiotensin-aldosterone system inhibitors, currently standard of care for heart failure, resulted in a modest reduction of cardiac fibrosis in several small clinical studies [25]. Their inability to fully abrogate the fibrotic response may be due in part to the pleiotropic and sometimes divergent roles of profibrotic mediators in different cell types. For example, SMAD3 activation in murine myofibroblasts played a protective role in cardiac injury by maintaining ECM network [17, 26], whereas SMAD3 signaling in cardiomyocytes promoted cardiomyocyte death and exacerbated systolic dysfunction after myocardial infarction (MI) [26]. Consequently, systemic inhibition of profibrotic pathways may produce both beneficial and detrimental effects.

Furthermore, cardiac fibroblasts play important homeostatic roles in the heart and contribute to the acute injury response by secreting ECM proteins in response to cardiomyocyte loss, facilitating scar formation and protecting the heart from rupture [18, 27••, 28]. Non-specific targeting of fibroblasts may therefore compromise the normal wound healing response and lead to untoward toxicities. Thus, highly specific targeting strategies in the design of therapeutic interventions against cardiac fibroblasts are needed.

Emerging evidence suggests that fibroblasts are not unidimensional cells whose sole function is to modulate ECM. Rather, they exhibit remarkable phenotypic heterogeneity [29, 30••] and mediate a diverse range of cellular functions including transducing proliferative and protective signals to cardiomyocytes [31, 32], clearing apoptotic cardiomyocytes [33], regulating electrical conduction [34, 35], and participating in inflammatory [36, 37] and angiogenic pathways [38]. This functional diversity is made possible by dynamic regulation of their gene expression profiles in response to mechanical stimuli, cytokines, or other mediators [32, 39, 40].

Taking advantage of this cellular diversity, the field has now begun to explore the feasibility of selectively targeting fibroblast subsets to reduce fibrosis. In a rodent model where up to 60% of periostin-expressing fibroblasts were ablated by diphtheria toxin, reduced cardiac fibrosis and improved heart function were observed following chronic Ang II infusion or MI [41•]. In these models, genetic recombination was driven by a tamoxifen-inducible *Cre*-transgene under the control of the *Postn* promoter [41•]. Importantly, ablation of periostin-expressing cardiac fibroblasts following MI did not compromise scar stability [41•]. In contrast, Kanisicak and colleagues generated a *Postn* knock-in strain containing the MerCreMer cassette and showed that all activated fibroblasts were labelled by periostin [27••]. In this model, total depletion of myofibroblasts using diphtheria toxin resulted in increased ventricular rupture and higher lethality [27••]. This inconsistency from the previous study is most likely due to differences in the genetic strategies as well as the level of cell depletion across the two approaches.

While diphtheria toxin–mediated ablation serves as a proof-of-principle to support selective targeting of fibroblast subpopulations to reduce fibrosis, this strategy is not therapeutically viable, as it requires multiple genetic recombinations to express the toxin gene construct in a defined cell type. Alternatively, cell therapy breakthroughs in the immuno-oncology field have made it possible to selectively deplete a defined cellular subpopulation for therapeutic purposes. In particular, by expressing a chimeric antigen receptor (CAR), cytotoxic T cells can be redirected to recognize specific antigens on cancer cells and thus mediate their killing [42]. It is not unreasonable to envision that similar strategies could be adopted for the selective depletion of non-cancer cells. Proof-of-principle of such a strategy has been achieved in the study by Aghajanian and colleagues [43••], who employed T cells engineered to express a CAR against fibroblast activation protein (FAP), a cell surface protein enriched in activated fibroblasts from patients with dilated or hypertrophic cardiomyopathy [44]. Using a murine model of hypertension induced by chronic infusion of Ang II/phenylephrine (PE), selective depletion of FAP^+^ fibroblasts in the heart by CAR T cell transplantation resulted in reduced fibrosis and improved cardiac function in treated animals [43••]. Importantly, whether ablation of activated fibroblasts would disrupt the normal wound healing response will need to be evaluated in future studies. Together, these studies suggest that targeting specific fibroblast subpopulations involved in pathological remodeling holds promise in limiting heart failure progression. A deeper understanding of fibroblast phenotypic heterogeneity, facilitated by recent advances in single-cell technologies, will enable segregation of fibroblast subtypes for more precise targeting of subpopulations enriched in disease states.

## Unraveling Fibroblast Transcriptomic Diversity Using Single-Cell Analyses

The last 10 years have seen the expansive development of single-cell transcriptomic technologies, enabling the characterization of transcriptional responses in subpopulations of cells in heterogenous tissues potentially masked by bulk RNA-seq approaches. A multitude of different scRNA-seq methods that vary at each step of the workflow have been developed, each with distinct advantages and disadvantages with regard to platform throughput, technology sensitivity, data coverage, and the per-cell cost [45, 46]. In general, all methodologies share a common workflow: single-cell isolation and capture, cell lysis, RNA reverse transcription, cDNA amplification, library preparation, sequencing, and computational analysis (reviewed in [47–49]). In parallel with the development of methods for single-cell isolation and RNA capture, a growing number of computational tools have also been developed (reviewed in [49, 50]), providing not only a bioinformatic pipeline for data quality control and batch normalization but also an unprecedented level of details into cellular heterogeneity and intercellular relationships.

Current high-throughput scRNA-seq technologies allow thousands of cells to be assayed simultaneously, enabling the identification of both novel and rare cell populations as well as the analysis of cell state transitions and complex intercellular communication networks [49] (Table 1). However, most high-throughput single-cell capture technologies have limits on cell size, precluding the accurate and unbiased selection of large cells like cardiomyocytes. Single-nucleus RNA-sequencing (snRNA-seq), where nuclear RNA species are sequenced instead of cytoplasmic ones, has addressed this challenge, allowing the simultaneous profiling of both cardiomyocytes and interstitial cells [51, 52••, 53••]. However, transcript detection sensitivity is reduced and isoform information is lost in snRNA-seq due to the lower abundance of RNA and enrichment of unspliced pre-mRNA in the nuclei [46]. Therefore, caution must be exercised when selecting a sequencing methodology for a particular experiment. In general, snRNA-seq may be sufficient for cell typing, whereas scRNA-seq could provide further information to facilitate target or biomarker identification beyond defining cellular heterogeneity [46].

**Table 1:** Advantages and disadvantages of current single-cell technologies

Method	Molecules assessed	Sample formats	Advantages	Disadvantages
Bulk RNA-seq	mRNA (or total RNA)	Fresh, frozen, or fixed tissue	Highest mRNA detection sensitivity	Tissue-wide assessment masks effects in cell subpopulations
scRNA-seq	mRNA	Fresh or methanol fixed single-cell suspension	Detection and characterization of rare cell subpopulations; Full-length RNA-seq protocols available for isoform-specific information	Cell dissociation-induced stress affects cell recovery and gene expression; Cell diameter limitations of microfluidics (platform specific)
snRNA-seq	pre-mRNA	Nuclei isolated from fresh or frozen tissue	Isolation of nuclei from tissue avoids cell dissociation-induced stress; Nuclei are not restricted by microfluidic diameter limitations	Decreased mRNA detection sensitivity relative to scRNA-seq; Enrichment of pre-mRNA masks isoform-specific information
CITE-Seq/REAP-seq	Surface protein and mRNA	Fresh single-cell suspension	Compatible with scRNA-seq methods; Low dropout rate of protein detection	Limited to surface proteins
Single-cell ATAC-seq	DNA	Nuclei isolated from fresh or frozen tissue	Assessment of gene regulatory regions; Characterization of transcription factor regulatory networks; Can be multiplexed with RNA-seq	Long distance interactions of open chromatin regions with genes not detectable; Low-density resolution of open chromatin footprints per cell
Spatial transcriptomics	Spatial mRNA assessment	Frozen or formalin-fixed paraffin-embedded tissue	Identification of spatial heterogeneity; Potential to identify localized cellular interactions	Most technologies capture multiple cells per spot

**Fig. 1:**
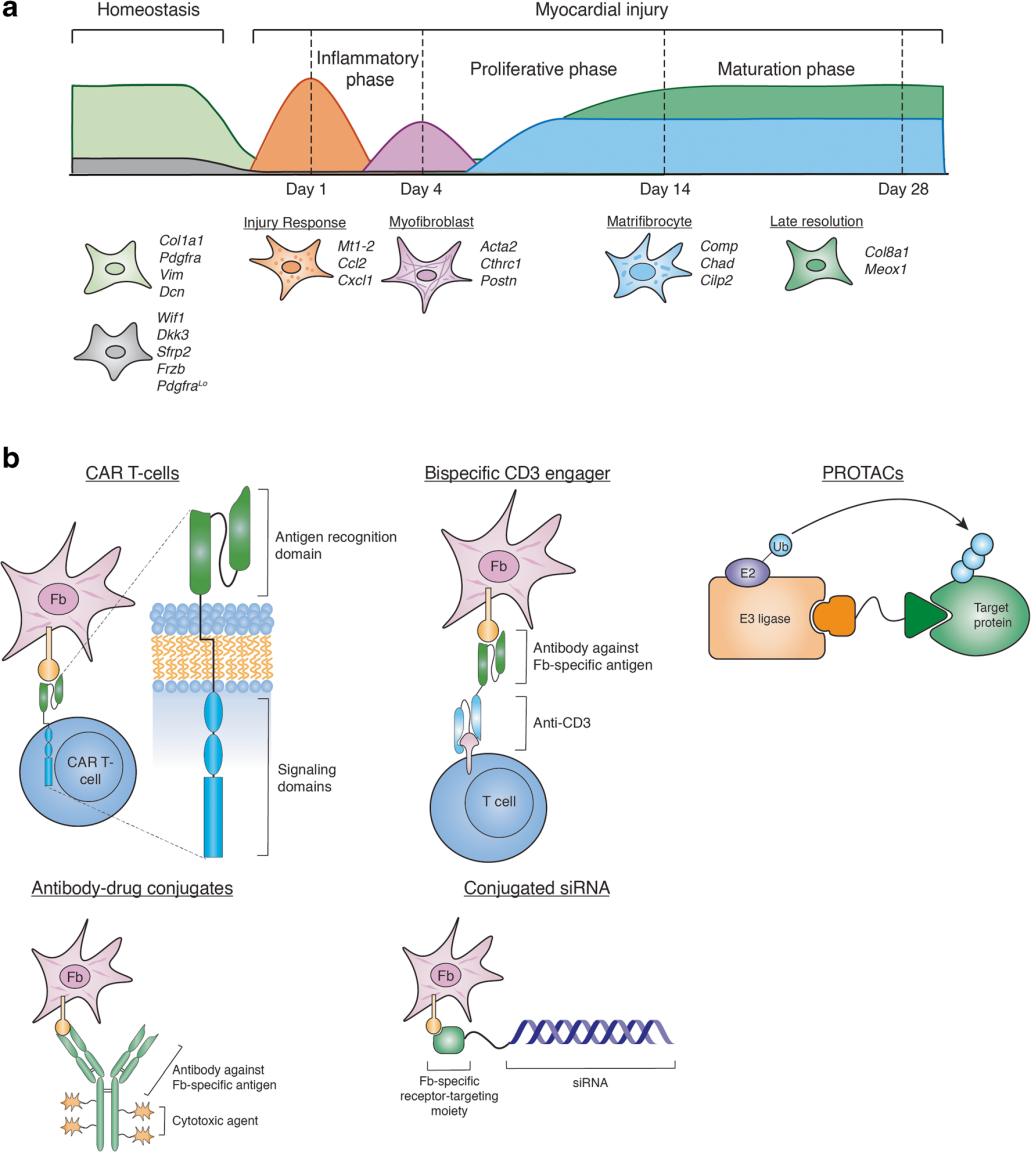
**a** Cardiac fibroblast dynamics in homeostasis and in response to myocardial injury revealed by scRNA-seq and snRNA-seq. Specific fibroblast subsets along with their marker genes are listed. **b** Examples of potential multispecific therapeutic drugs targeting cardiac fibroblasts (Fb).

Taking advantage of single-cell transcriptomic technologies, studies in cardiac interstitial cells have revealed remarkable phenotypic plasticity of cardiac fibroblasts, whose transcriptional profile is dynamically regulated in response to external stimuli (Table 2 and Fig. 1a).

**Table 2:** Single-cell or single-nucleus RNA-seq studies on cardiac fibroblasts

Publication	Publication year	Species/reporter strain	Tissue	Cell type	Context/model	scRNA-seq methodology	Total number of cells analyzed
Kanisicak et al. Nat Commun [27••]	2016	Mouse (*Tcf21*^*MCM/+*^; *Rosa26-EGFP* and *Postn*^*MCM/+*^; *Rosa26-eGFP*)	Ventricles	Non-CM	1 week after MI	Smart-seq; scRNA-seq	185
DeLaughter et al. Dev Cell [54]	2016	Mouse	LV, RV, and LA	CM and non-CM	E9.5 to P21	Smart-seq; scRNA-seq	>1200
Li et al. Dev Cell [55]	2016	Mouse	IFT, RA, LA, AVC, RV, RS, LS, LV, OFT, PO, and DO	CM and non-CM	E8.5, E9.5, and E10.5	Smart-seq; scRNA-seq	2233
Schafer et al. Nature [56]	2017	Mouse	LV	Non-CM	Control and *Pln*^*R9C/+*^	Chromium (10x Genomics); scRNA-seq	-
Gladka et al. Circulation [57]	2018	Mouse	LV	CM and non-CM	3 days after I/R	SORT-seq; scRNA-seq	932
Hu et al. Genes Dev [51]	2018	Mouse	Ventricles	CM and non-CM	Control and *ERRα*^*-/-*^*;Myh6Cre*^*+*^; *ERRγ*^*fl/fl*^; P9 to P10	sNucDrop-seq; snRNA-seq	7760 for control, 7323 for knockout
Skelly et al. Cell Rep [58•]	2018	Mouse	LV	Non-CM	10 weeks of age; males and females	Chromium (10x Genomics); scRNA-seq	10,519
Kretzschmar et al. Proc Natl Acad Sci U S A [59]	2018	Mouse (*Mki67*^*tagRFP*^)	Ventricles	CM and non-CM	1 week old, 8-10 weeks old; 14 days after MI or I/R	SORT-seq; scRNA-seq	1939
Xiao et al. Genes Dev [60]	2019	Mouse	Whole heart	Non-CM	Control and *Tcf21*^*iCre*^; *Lats1*^*fl/fl*^; *Lats2*^*fl/fl*^; *Rosa26*^*mTmG*^	Drop-seq; scRNA-seq	17,501
Farbehi et al. Elife [61]	2019	Mouse (*Pdgfra*^*GFP/+*^)	Whole heart	Non-CM	3 and 7 days after MI	Chromium (10x Genomics); scRNA-seq	13,331
Cui et al. Cell Rep [62]	2019	Human	LV, RV, LA, RA, TV, MV, AV, PV, AO, PO, and IS	CM and non-CM	From 5 to 25 weeks of gestation	SRTR-seq; scRNA-seq	4948
McLellan et al. Circulation [63]	2020	Mouse	Whole heart	CM and non-CM	2 weeks after Ang II	Chromium (10x Genomics); both scRNA-seq and snRNA-seq	29,558
Ren et al. Circulation [64]	2020	Mouse	Whole heart	CM and non-CM	2, 5, 8, 11 weeks after TAC	ICELL8; scRNA-seq	11,492
Ruiz-Villalba et al. Circulation [65]	2020	Mouse (*Col1a1-GFP*)	Ventricles	Fibroblasts	7, 14, 30 days after MI	Chromium (10x Genomics); scRNA-seq	29,176
Forte et al. Cell Rep [66•]	2020	Mouse (*Wt1*^*Cre*^; *RosaZsGreen*^*fl/+*^)	Ventricles	non-CM	1, 3, 5, 7, 14, and 28 days after MI	Chromium (10x Genomics); scRNA-seq	36,847
Wang et al. Nat Cell Biol [67]	2020	Human	LV, RV and LA	CM and non-CM	Healthy adult (16; 2 women, 14 men), HF (6 men) and HF treated with LVAD (2 men)	ICELL8; scRNA-seq	21,422
Litviňuková et al. Nature [52••]	2020	Human	LV, RV, LA, RA, LV apex, and IS	CM and non-CM	Healthy adult (7 women, 7 men)	Chromium (10x Genomics); both scRNA-seq and snRNA-seq	45,870 cells, 78,023 CD45+ cells and 363,213 nuclei
Tucker et al. Circulation [53••]	2020	Human	LV, RV, LA, and RA	CM and non-CM	Healthy adult (4 women, 3 men)	Chromium (10x Genomics); snRNA-seq	287,269 nuclei
Wang et al. Cell Rep [68]	2020	Mouse	Ventricles	CM and non-CM	1 and 3 days after MI at P1 and P8	Chromium (10x Genomics); scRNA-seq	17,320
Alexanian et al. Biorxiv [40]	2020	Mouse	Ventricles	non-CM	TAC and treatment with BET-inhibitor JQ1	Chromium (10x Genomics); scRNA-seq	35,551

*Abbreviations*: *LV*, left ventricle; *RV*, right ventricle; *LA*, left atrium; *RA*, right atrium; *IFT*, inflow tract; *AVC*, atrioventricular canal; *RS*, right ventricular septum; *LS*, left ventricular septum; *OFT*, outflow tract; *PO*, proximal outflow tract; *DO*, distal outflow tract; *TV*, tricuspid valve; *MV*, mitral valve; *AV*, aortic valve; *PV*, pulmonary valve; *AO*, aorta; *PO*, pulmonary artery; *IS*, interventricular septum; *TAC*, transverse aortic constriction; *MI*, myocardial infarction; *I/R*, ischemia reperfusion; *HF*, heart failure; *LVAD*, left ventricular assist device

### Fibroblast Subtypes in Homeostasis

Fibroblast-like cells can be detected as early as 11.5 days post-coitum in the developing mouse heart, and these embryonic cells express canonical fibroblast genes such as *Col1a1* and *Dcn* [51, 54]. This coincides with the formation of the epicardium, a mesothelial layer that migrates from the pro-epicardial organ to form a sheath of cells covering the myocardial wall [69]. From 12.5 days post-coitum, the epicardium undergoes epithelial-to-mesenchymal transition to generate interstitial fibroblasts and smooth muscle cells [69]. In both mouse and human hearts, the proportion of fibroblast-like cells was shown to increase throughout embryonic development [54, 55, 62]. Analysis of cell cycle–related genes and gene regulatory networks revealed that a major wave of fibroblast proliferation occurred during early gestation [62]. These fibroblasts gradually became more mature, accompanied by an upregulation of ECM protein expression [62]. Within the adult heart, lineage tracing suggested their continuous cellular turnover even under homeostatic conditions [59].

In the adult human heart, fibroblasts have been shown to constitute about 15–30% of all cells, with regional differences between atrial and ventricular tissues [52••, 53••]. This percentage is somewhat higher than what has been described for mouse cardiac fibroblasts, which account for less than 20% of non-myocytes under homeostatic conditions [70•]. Notably, this is not a homogeneous population of cells. In fact, single-cell analysis has provided a glimpse into the remarkable transcriptional heterogeneity of the resident fibroblast population. For example, subclustering of fibroblasts in the adult murine heart identified two transcriptionally distinct subpopulations [58•, 61, 63, 65] (Fig. 1a). Both populations expressed *Col1a1*, but one of them was characterized by low abundance of the canonical fibroblast markers *Pdgfra* and *Tcf21* [58•, 61]. This distinct subpopulation instead was enriched in Wnt signaling pathway genes such as *Wif1*, *Dkk3*, *Sfrp2*, and *Frzb* [61, 63, 66•]. Their origin and function in the adult heart remain a subject of debate [61, 66•]. Unsupervised clustering of adult human cardiac fibroblasts has also identified transcriptionally distinct subpopulations. Combining scRNA-seq and snRNA-seq data from human organ donors without overt cardiovascular disease, Litviňuková and colleagues [52••] identified a subcluster of stromal cells displaying characteristics of activated fibroblasts, including expression of *POSTN* and *FAP*. Fibroblast activation in this context may result from age-related changes in cardiac physiology that leads to progressive fibrotic remodeling, or from responses to cardiac stress that were not diagnosed clinically. Similar populations were absent in an independent study using only snRNA-seq on samples collected from younger donors [53••]. This discrepancy may be attributed to differences in the health status of the donors, the more detailed clustering in Litviňuková et al., or both. Notably, in both studies, fibroblasts display chamber-specific distributions across the heart, likely related to their diverse developmental origins and specialized functions [52••, 53••]. Together, these single-cell analyses of the healthy human heart provide an information-rich reference to deepen our understanding of cardiac physiology in homeostatic conditions.

Using available repositories of curated ligand-receptor pairs [71, 72], single-cell transcriptomic data have enabled the analysis of intercellular communication networks in heterogeneous tissues such as the heart. Employing these computational tools, scRNA-seq studies have identified dense connections between fibroblasts and multiple cardiac cell types [58•, 67]. Indeed, fibroblasts were shown to be the most trophic cell population, with multiple signaling circuits supporting the survival of other cardiac cells such as pericytes, endothelial cells, and mural cells [58•, 63]. These intercellular communication networks are disturbed in response to injury, coinciding with initiation of cardiac remodeling [64, 67]. These analyses further corroborate the diverse and essential role of the resident fibroblasts in maintaining cardiac homeostasis.

### Fibroblast Subtypes in Heart Injury

In response to myocardial injury such as ischemic, mechanical, or inflammatory stress, damaged cardiomyocytes undergo apoptosis and secrete cytokines and soluble factors to trigger inflammatory infiltration and fibroblast activation [2, 4]. Activated fibroblasts are characterized by increased proliferation, ECM synthesis, expression of contractile proteins, and cytokine/growth factor secretion [2, 4]. Traditionally, detection of activated fibroblasts relied on expression of α-smooth muscle action (αSMA, *ACTA2*), a contractile protein whose presence defines the myofibroblast state [73]. However, emerging evidence suggests that not all activated fibroblasts express αSMA [27••, 41•, 74], and in fact, myofibroblasts are only a portion of the matrix-producing fibroblasts that underlie the fibrotic response [75••]. Hence, the field needs more reliable and definitive means to accurately profile fibroblasts after injury.

Independent studies by Fu et al. [75••] and Ivey et al. [76] using lineage tracing and reporter strains to label resident fibroblasts showed that fibroblast proliferation peaked within the first week after MI, with a 3-5-fold expansion in cell number. Cell cycle entry was confirmed at the transcriptional level by subsequent scRNA-seq analyses [61, 65, 66•, 68]. While Fu et al. [75••] observed minimal cell proliferation in regions outside of the infarct, other studies have presented contrasting evidence, demonstrating global activation of a proliferative program in the resident fibroblasts [66•, 76]. A similar temporal pattern of proliferation was observed in additional models of cardiac injury induced by TAC or isoproterenol injections, with peak proliferation occurring around the first week after injury and rapidly returning to baseline by 2 weeks [76].

Coinciding with the proliferative phase, expression of genes associated with fibroblast activation such as *Acta2*, *Postn*, and *Tnc* was detected 3 days after MI, suggesting that fibroblasts rapidly adopt an activated phenotype in response to injury [57, 61, 66•, 75••] (Fig. 1a). Notably, inclusion of multiple closely spaced timepoints immediately after injury allowed Forte and colleagues [66•] to uncover a novel fibroblast subpopulation appearing as early as 1 day after MI, which was termed “injury response” fibroblasts (Fig. 1a). This subcluster expressed high levels of metallothioneins *Mt1-2*, monocyte-macrophage chemoattractants *Ccl2*, *Ccl7*, and *Csf1*, and neutrophil activators *Cxcl1* and *Cxcl5*, implicating this early fibroblast state in the initiation of the inflammatory response [66•]. A similar subcluster was also identified following MI injury in the post-natal day 8 heart, when the murine heart loses regenerative capacity [68]. By cellular trajectory analysis, these injury response cells were shown to quickly evolve into myofibroblasts by day 3, accompanied by expression of markers such as *Acta2*, *Fn1*, and *Cthrc1* [66•] (Fig. 1a), highlighting the transcriptional plasticity of fibroblasts and the rapidly changing cellular landscape in the injured myocardium.

The fate of activated fibroblasts has also been a subject of extensive investigation. In models of reversible injury such as Ang II/PE, withdrawal of the injury stimuli was shown to cause dedifferentiation of myofibroblasts, with reduction in cell cycle genes and loss of αSMA expression [27••]. In humans, left ventricular assist device implantation and unloading of the heart was also associated with transcriptional normalization of the cardiac fibroblasts, which correlated with recovery of cardiac function [67]. With permanent injury such as MI, newly formed fibroblasts persisted in the infarct region and were not turned over [75••]. Nevertheless, myofibroblasts seemed to be a transient differentiated state, as αSMA expression was nearly undetectable by 2 weeks after injury [66•, 75••]. Instead, another unique differentiated state was shown to predominate the maturation phase [75••] (Fig. 1a). Termed matrifibrocytes, this fibroblast subset was closely related to myofibroblasts, but exhibited reduced contractile and secretory properties by gene expression analysis [66•]. They were enriched in genes associated with bone and cartilage remodeling, such as *Comp*, *Chad*, and *Cilp2*, consistent with the development of a stable, chondrogenic-like collagenous scar [66•, 75••]. A second fibroblast subset prevalent in the maturation phase was termed “late resolution” fibroblasts (Fig. 1a). They were proposed to arise from chronic pathological remodeling and expressed genes regulating TGFβ activity and ECM components, in line with continued dynamic remodeling of the myocardium during chronic injury [66•].

Presence of matrifibrocyte-like cells has been suggested in other murine models of cardiac fibrosis and heart failure such as chronic Ang II infusion [63]. However, in this study, a distinct population of *Acta2*^*+*^ myofibroblasts was not identified [63]. This may be explained by the choice of a single timepoint after initiation of Ang II (i.e., 2 weeks), which may have precluded the detection of early transcriptional changes in the fibroblast population. In an independent study by Ruiz-Villalba et al. [65], a distinct fibroblast subpopulation defined by markers including *Cthrc1*, *Ddah1*, *Fmod*, and *Comp* was dynamically upregulated in response to both MI and chronic Ang II infusion. Similar to matrifibrocytes, this population was characterized by gene ontology terms related to ECM assembly and collagen fibril organization [65]. However, in contrast to matrifibrocytes which persisted in the injured myocardium [66•, 75••], the relative enrichment of this subpopulation was transient, peaking at 2 weeks post-injury and then decreasing under more chronic conditions [65]. Mice deficient in *Cthrc1* displayed an impaired fibrotic response and susceptibility to ventricular rupture after MI, indicating an important role for this population in the wound healing response, hence the term “reparative cardiac fibroblasts” [65]. Whether these cells represent an intermediate phenotype between myofibroblasts and matrifibrocytes remains to be investigated. Furthermore, one important question to address with regard to matrifibrocytes is the degree to which they contribute to pathological remodeling and heart failure progression, as this will shed light on whether specific targeting of this fibroblast subpopulation represents a therapeutically viable option to treat cardiac fibrosis. Another key consideration is the extent to which these observations made in murine models could translate into humans. A full spectrum of single-cell analysis of the human heart in different disease conditions, integrated with the information on the diverse subgroups of fibroblasts found in animal models, are critical for understanding the role of cardiac fibroblasts in human heart failure and for future therapeutic development.

### Additional Applications of Single-Cell Technologies

In addition to scRNA-seq, numerous next-generation sequencing applications have made it possible to profile other biomolecules or cellular processes in a heterogeneous tissue at the single-cell level [49] (Table 1). For example, single-cell sequencing of transposase-accessible chromatin (scATAC-seq) and single-cell chromatin immune-precipitation sequencing (scChIP-seq) reveal genome-wide chromatin organization and accessibility, providing insight into gene regulatory landscapes that govern transcription [77, 78]. A recent study employing scATAC-seq identified dynamic and reversible changes in chromatin accessibility of cardiac fibroblasts that correlated with cardiac disease severity and demonstrated the plausibility of targeting transcriptional switches to reverse cardiac fibrosis [40]. Global changes to chromatin accessibility were also evident in the neonatal mouse heart after MI injury, especially in non-regenerative hearts at post-natal day 8, suggesting MI-induced transcriptional changes coinciding with cardiac remodeling [68]. Together these data demonstrated the plasticity of cardiac fibroblasts at the level of epigenetic regulation.

Another emerging technology, spatial transcriptomics, retains information on local tissue context [79, 80]. The microenvironment in which a fibroblast resides, including its surrounding ECM and neighboring cells, can have a strong influence on its phenotype and activation status [12, 39, 81]. This is an important but perhaps underappreciated aspect of fibroblast biology. Spatial transcriptomics together with the aforementioned analysis of intercellular communication networks can help depict a clearer picture of how stressed cardiomyocytes signal to recruit and activate immune cells and fibroblasts, and how fibroblasts crosstalk with other cell types to influence cardiac remodeling. Availability of spatial information will also make it possible to correlate transcriptional findings with the extent of tissue remodeling in the failing heart.

Cellular indexing of transcriptomes and epitopes by sequencing (CITE-seq) and RNA expression and protein sequencing assay (REAP-seq) are yet another variation of scRNA-seq. These techniques use oligonucleotide-barcoded antibodies to measure protein expression concurrently with cellular transcripts [82, 83]. These methodologies have been leveraged to study immune cell heterogeneity [84], and conceivably, will be helpful in providing enhanced granularity of fibroblast subsets. CITE-seq and REAP-seq are currently limited to the detection of prespecified cell surface proteins with corresponding antibodies, whereas the nascent field of single-cell proteomics with enhanced sensitivity and throughput [85] holds promise for a full integration of multimodal single-cell omics. Simultaneous interrogation of multi-level cellular regulatory mechanisms will provide a holistic view of individual cells, from upstream gene regulatory networks to downstream spatially resolved phenotypes such as protein expression.

Undoubtedly, results of these single-cell studies and associated computational analyses will need to be validated experimentally to confirm the expression profiles of genes of interest, the cellular composition of the tissue being studied, and cell state transitions in response to external stimuli. Presence of specific cellular subtypes will also need to be validated functionally, which could be achieved by genetic manipulation of one or more marker genes. Compared with bulk RNA-seq, scRNA-seq generally has lower sequencing depth, limiting its sensitivity to detect low-expressing genes and the ability to discriminate true biological signal from technical noise [86]. Notwithstanding their limitations, scRNA-seq technologies have revealed specialized fibroblast populations that are of potential therapeutic interest at an unprecedented level of resolution. This rich information will inform development of novel treatments targeting cardiac fibroblasts.

## Novel Therapeutic Strategies for Targeting Cardiac Fibroblasts

The success of targeting pathogenic fibroblasts to achieve disease amelioration in preclinical models has pointed to a novel treatment paradigm for heart failure. Nevertheless, given the wide distribution of fibroblasts and lack of specific markers to label them [4, 6], how to achieve therapeutic specificity without inadvertent targeting of other cardiac cell types and/or other tissue fibroblasts becomes a major hurdle in drug development. Innovations in the biopharmaceutical industry over the last two decades have provided a potential solution, with the development and commercialization of multispecific drugs. In contrast to the classical “one target and one drug” approach where a molecule binds to a specific target and modulates its function, multispecific therapeutics work by engaging two or more entities (reviewed in [87]) (Fig. 1b). As a result of this engagement, a therapeutic agent can be localized to the desired site of action; alternatively, a target can be brought into close proximity of an endogenous effector [87]. These powerful capabilities have the potential to transform medicine with extraordinary precision, enabling access to biological entities previously considered intractable.

Currently, most commercialized multispecific agents are approved for oncology or hematology indications [87], but their utilization in other therapeutic areas is rapidly emerging. For example, a proof-of-concept study illustrated the feasibility of targeting cardiac fibroblast subsets using T cells engineered to express a CAR against FAP [43••]. A CAR is comprised of an extracellular antigen-binding domain, typically a single-chain variable fragment derived from an antibody, and an intracellular signaling domain derived from the T cell receptor ζ chain and costimulatory domains such as CD28, 4-1BB, or OX40 [42] (Fig. 1b). This construction programs T cells to recognize a specific protein expressed on the surface of target cells, leading to their cytotoxic killing [42]. FAP, expressed specifically by stromal cells, is a cell surface protein induced at sites of active ECM turnover [44], making it a desirable target for depleting activated fibroblasts without affecting other cardiac cells and quiescent fibroblasts. However, what percentage of FAP^+^ cells was depleted by CAR T cells and to what extent FAP^+^ cell depletion was required for amelioration of fibrosis was not reported in this study. In addition, whether non-specific actions of the modified T cells could contribute to the observed phenotype remains unclear. Nevertheless, this study represents an important milestone in the advancement of cardiovascular medicine beyond traditional small molecules and biologics. Subsequent attempts at utilizing CAR T cells to reduce tissue fibrosis targeted the cell surface protein urokinase-type plasminogen activator receptor (uPAR), whose expression is induced in senescent cells [88]. uPAR-specific CAR T cells improved senescence-associated liver fibrosis induced by carbon tetrachloride or a high-fat diet by targeting both macrophages and hepatic stellate cells [88].

CAR T cell immunotherapy is one example of multispecific “matchmakers” that work by inducing proximity, in this case, between a target cell type and effector T cells. Bispecific CD3 engagers represent another strategy that redirects T cell activity towards a defined cell population. As the name implies, these are bispecific antibody–based formats with one domain that binds a surface antigen on target cells and a second domain that binds the CD3 subunit of T cell receptor, leading to T cell activation and lysis of the target cells [89] (Fig. 1b). Recently, bispecific antibody–based biologics that redirect natural killer cells have also been developed [90]. These matchmakers have great therapeutic potential because they harness an endogenous biological mechanism for effector function rather than having to directly modulate the target [87]. As a result, most biological entities such as macromolecules, organelles, and even cells can be targeted with high specificity. Nevertheless, in addition to the inherent challenges faced by multispecific therapeutics (reviewed in [87]), successful targeting of cardiac fibroblasts requires knowledge of the precise expression patterns of the target cell surface antigens, as any on-target killing of cells other than the intended fibroblast subpopulation(s) poses safety risks that are unlikely to be acceptable for a cardiovascular disease indication.

An exciting class of matchmakers mobilizes endogenous molecular machinery to achieve targeted degradation of proteins, nucleic acids, and even organelles (reviewed in [91]). Examples include the heterobifunctional proteolysis-targeting chimeras (PROTACs), which are chimeric small molecules with one module binding the target protein and the other binding a ubiquitin ligase (Fig. 1b). The induced proximity between the two leads to ubiquitylation of the target protein followed by its degradation in the proteasome [92]. Though still in its infancy, targeted degradation as a therapeutic modality has garnered considerable momentum in the industry due to its versatility, effectiveness in abolishing all functions of a target, and potentially long-lasting effect [91].

In addition to matchmakers, another class of multispecific therapeutics termed “tetherbodies” enrich a drug at the desired site of action, thus reducing side effects in off-target tissues [87]. Examples include antibody-drug conjugates and RNA-based therapeutics targeted for a specific tissue. Current antibody-drug conjugates in development are designed for oncology indications, taking advantage of tumor-specific cell surface antigens that concentrate a cytotoxic drug at tumor cells (reviewed in [93]) (Fig. 1b). A similar strategy targeting cardiac fibroblasts for cytotoxic killing may be employed. Alternatively, cell-specific inhibition of profibrotic pathways such as TGFβ signaling could be realized with a bispecific format, using a TGFβ inhibitor as one arm and an antibody against a fibroblast-specific antigen as the other arm. Development of tetherbodies for enrichment of therapeutics at the site of action has been hampered by the paucity of information on tissue-restricted cell surface receptors. Knowledge gained from multi-omics studies at the single-cell level may provide a breakthrough and greatly expand the utility of these agents.

RNA-based therapeutics such as small-interfering RNA (siRNA), antisense oligonucleotide (ASO), modified mRNA and microRNA mimetics greatly expand the domain of druggable targets while offering the advantages of ease of sequence-based design and high specificity (reviewed in [94, 95]). They offer vast opportunities for novel forms of therapeutic mechanisms that are difficult to achieve with traditional small molecules and biologics, such as upregulating or repressing gene expression, altering mRNA splicing, and targeting non-coding RNAs [96]. Currently, the most successful targeting approach for oligonucleotide therapies involves chemical conjugation to ligands for the hepatocyte-specific asialoglycoprotein receptor, resulting in uptake by the liver [97, 98]. Additional carriers are actively being explored to enhance transmembrane delivery into extrahepatic tissues, although none has reached clinical development. Recently, using fatty acids such as docosanoic acid and myristic acid as carriers, researchers were able to achieve 30–45% gene knockdown in the heart [99, 100]. However, to mediate uptake by defined cell types such as the cardiac fibroblast, a highly specific receptor-targeting moiety such as a ligand, aptamer, or antibody would be more desirable as a carrier (Fig. 1b). Proof-of-concept studies in animals have demonstrated this potential, revealing efficient gene knockdown with conjugated siRNA in defined cell types including activated lymphocytes [101], macrophages [102], or tumor cells [102, 103].

Multispecific therapeutic agents rely on target tissue-enriched proteins to achieve specificity; however, it is unlikely that a unique marker is only expressed specifically in cardiac fibroblasts [6]. Inadvertent targeting of other tissues would narrow the therapeutic window. To address this challenge, inspiration could be drawn from recent innovations in the oncology field. For example, dual-receptor CAR T cells have been designed where a synthetic Notch receptor for one antigen induces the expression of a CAR for a second antigen [104, 105]. These T cells are only activated in the presence of both antigens, thus enhancing discrimination between target cells and “bystander” cells [104, 105]. Another strategy involves masking the antigen-binding site of an antibody therapeutic with a peptide, which gets cleaved by proteases specifically found in the tumor microenvironment [106].

## Conclusion

Thanks to advances in genetic manipulation and scRNA-seq technologies, the field of cardiac fibroblast biology is entering a new era, with ever more recognition for the diverse roles these cells play in cardiac physiology. Our current understanding of cardiac fibroblasts is based primarily on genetic manipulation within animal models of disease, and it is critical that findings from these studies be validated in humans. Recent scRNA-seq studies in the healthy human heart are encouraging and will serve as valuable resources in this regard [52••, 53••, 67]. Further leveraging of single-cell technologies to identify and characterize pathological states of fibroblasts in the setting of human heart failure will be critical to a better understanding of their contribution to disease progression and to development of novel treatment strategies.

With multispecific drugs significantly expanding the therapeutic toolbox, specific targeting of tissue fibroblasts may soon become a reality. However, multispecific therapeutics are inherently more complex than traditional small molecules or biologics. For them to realize their full potential, challenges specific to multispecific formats must be considered early in their development [87]. Taken together, technological advances are revolutionizing the way we study human diseases. In addition, we anticipate that they will aid in the development of novel and effective therapeutic strategies to target cardiac fibroblasts, a key player in cardiovascular disease.
